# Antibacterial quaternary ammonium chitosan/carboxymethyl starch/alginate sponges with enhanced hemostatic property for the prevention of dry socket

**DOI:** 10.3389/fbioe.2022.1083763

**Published:** 2023-01-09

**Authors:** Xuyang Deng, Danyang Wang, Dongjie Zhang, Ming Sun, Liying Zhou, Yuxi Wang, Xiaowen Kong, Changqing Yuan, Qihui Zhou

**Affiliations:** ^1^ Department of Stomatology, The Affiliated Hospital of Qingdao University, Qingdao University, Qingdao, China; ^2^ School of Stomatology, Qingdao University, Qingdao, China; ^3^ Institute for Translational Medicine, The Affiliated Hospital of Qingdao University, Qingdao University, Qingdao, China; ^4^ Department of Gastroenterology, The Affiliated Hospital of Qingdao University, Qingdao, China; ^5^ Dental Biomaterials Technology Innovation Center of Qingdao, Qingdao, Shandong, China; ^6^ School of Rehabilitation Sciences and Engineering, University of Health and Rehabilitation Sciences, Qingdao, China; ^7^ Zhejiang Engineering Research Center for Tissue Repair Materials, Wenzhou Institute, University of Chinese Academy of Sciences, Wenzhou, China

**Keywords:** marine polysaccharide, bio-multifunctional sponge, antibacteria, rapid hemostasis, tooth extraction, dry socket

## Abstract

Tooth extraction commonly leads to postoperative wound bleeding, bacterial infection, and even the occurrence of dry socket. Therefore, developing a biomedical material with favorable antibacterial and excellent hemostatic properties to prevent the post-extraction dry socket is necessary. Herein, quaternary ammonium chitosan/ carboxymethyl starch/alginate (ACQ) sponges are developed *via* Ca^2+^ cross-linking, electrostatic interaction, and lyophilization methods. The results show that the bio-multifunctional sponges exhibit interconnected porous structures with significant fluid absorption rates and suitable water vapor transmission rates. *In vitro* cellular and hemolysis experiments indicate that the developed sponges have acceptable biocompatibility. Notably, the constructed sponges effectively inhibit the growth of *E. coli*, *S. aureus,* and *C. albicans*, as well as achieve rapid hemostasis in the mouse liver injury and mini-pig tooth extraction models by absorbing blood and promoting red blood cell adhesion. Thus, the created bio-multifunctional sponges show tremendous promise as a hemostatic material for wound management after tooth extraction.

## 1 Introduction

In oral surgery, tooth extraction is a general treatment that frequently results in severe trauma. The elderly, especially patients on anticoagulants, have difficulty forming blood clots and often experience uncontrollable bleeding after dental extraction (Inokoshi et al., 2021). And dry socket would occur when the blood clot is removed or dissolved too early to expose the alveolar bone (Catanzano et al., 2018). Dry socket can cause sharp pain that affects the patient’s chewing and swallowing, making oral hygiene difficult to maintain (Yan et al., 2022). Absorbable gelatin sponges (AGS) with hemostatic properties have been employed to avoid dry socket in tooth extraction treatment (Wang et al., 2023). However, AGS lacks antimicrobial components and may cause an allergic reaction in the body, leading to inflammation at the wound site and increasing the rate of infection (Wei et al., 2020). Additionally, some biomaterials, such as platelet-rich fibrin (PRF) have also been used in clinical treatment. However, PRF is difficult and expensive to prepare and has insufficient hemostatic capacity, which limits its application to tooth post-extraction treatment (Otake et al., 2021). Therefore, it is highly desirable to develop a hemostatic material with good biocompatibility, low cost, and antibacterial properties for managing socket wounds and preventing dry socket following tooth extraction.

Sponge materials that present 3D network structures have developed rapidly in recent years (Jesionowski et al., 2018; Yuan et al., 2022; Wang et al., 2023). The porous network with high absorptive properties could serve as a physical barrier at the injured site to absorb exudate, maintain a moist microenvironment and promote wound healing (Wan et al., 2020; Yang E. et al., 2022). Meanwhile, this effective and rapidly absorbed effect may also lead to an enrichment of endogenous blood coagulation factors at the site of injury, thus promoting thrombosis and ultimately hemostasis (Ghobril and Grinstaff, 2015). Natural sodium alginate (AG) with good biocompatibility and biodegradability is commonly used as a matrix material for sponges (Hao et al., 2021; Xie et al., 2022b). AG could absorb water several times its weight and also promotes adhesion between the composite and the wound, making it a desirable material for hemostasis (Wang Y. et al., 2021; Lv et al., 2021; Xie et al., 2022a). Starch-based hemostatic agents belong to the class of passive hemostatic agents and exhibit good biodegradability and biocompatibility (Panwar et al., 2019). Starch granules could increase the concentration of platelets and endogenous clotting factors at the bleeding site by absorbing fluid from the blood, thus producing an effective hemostasis effect (Antisdel et al., 2009). Carboxymethyl starch (CMS) is an anionic polysaccharide that is soluble in water at room temperature, and as a derivative of starch, it has been widely used in wound healing and regenerative medicine (Chen et al., 2018). Carboxymethylation of starch increases the hydrophilicity, thereby increasing the fluid absorption of starch and further improving hemostatic efficiency (Massicotte et al., 2008). Therefore, CMS is often used with other hemostatic materials to enhance the hemostatic effect.

As the only naturally derived alkaline polysaccharide with a positive charge, CS has numerous beneficial characteristics, including satisfied biodegradability, biocompatibility, hemostatic capabilities, and antibacterial properties, as well as the promotion of wound healing (Yu et al., 2019; Wang Z. et al., 2021; Hao et al., 2022b; Xu N. et al., 2022). However, the application of CS is limited by poor water solubility (Zhao et al., 2017). It was found that the modification of CS with quaternary ammonium salts can significantly improve water solubility while enhancing antimicrobial properties (Zhao et al., 2018; Liang et al., 2020; Zong et al., 2022). Moreover, the enhanced positive charge of quaternary ammonium chitosan (Qch) is thought to improve the hemostatic ability of CS, which attracts and interacts with large numbers of red blood cells through electrostatic action (Huang et al., 2017). A positive charge-based procoagulant effect like this has also accelerated the development of new hemostatic materials with cationic properties (Behrens et al., 2014). Inspired by the observations above, AG/CMS/Qch (ACQ) composite sponge would be an excellent candidate as a bio-multifunctional socket dressing, which has not been reported.

In the present study, a series of composite sponges based on AG were prepared through electrostatic interaction, Ca^2+^ crosslinking, and lyophilization methods. The physicochemical features of the composite sponges were measured by fourier transform-infrared (FTIR) spectra, scanning electron microscopy (SEM), pore size, porosity, fluid absorption, and water vapor transmission rate (WVTR). The interaction between materials and cells was also detected *in vitro* to evaluate the biocompatibility of sponges. The antibacterial performances, as well as the *in vitro* and *vivo* hemostatic properties of the sponges, were systematically characterized. Our results indicate that the use of bio-multifunctional ACQ compound sponges after tooth extraction holds out a lot of potential for hemostasis and the avoidance of dry socket.

## 2 Materials and methods

### 2.1 Synthesis of Qch

The CS powder (Mw = 300 kDa, Golden-Shell, China) was dissolved in an amalgamated solution made up of KOH, LiOH-H_2_O, urea (Sinopharm, China), and double distilled water (DDW) with a weight ratio of 7:8:8:77. To create a clear solution, the mixture was frozen at -20°C for an entire night before being thawed and mixed at 5°C. 3-Chloro-2-hydroxypropyl trimethyl ammonium chloride (Sinopharm, China) was then added dropwise to the CS solution. After adjusting the pH with sufficient HCl solution (Sinopharm, China), the mixture was dialyzed with DDW for a week. The solution was freeze-dried using a lyophilizer (Christ, Germany) to get pure Qch powder.

### 2.2 Synthesis of ACQ composite sponges

Briefly, the AG (M/G = 1.2, Mw = 600 Kg/mol, the viscosity is 700 mPa s, Qingdao Bright Moon Seaweed Group Co., Ltd, China) was dissolved in DDW (2 wt%), stirred at room temperature for 4 h, and filtered 3 times with a pressure pump. The AG solution was then poured into 24-well plates and lyophilized to form AG sponges. The freeze-dried sponges were cross-linked with a mixture of glycerol, CaCl_2,_ and anhydrous ethanol in the ratio of 3:7:90 by weight for 6 h, rinsed with excess DDW, and lyophilized again to fabricate the final AG sponges (Hao et al., 2020). The rest of the sponges were synthesized in a similar way to AG sponges. AC sponges were made by dissolving 1 g AG and 1 g CMS in 66.66 mL DDW. For ACQ-1 sponges, 1 g AG, 1 g CMS and .4 g Qch were dissolved in 70.66 mL DDW. ACQ-2 sponges were obtained by dissolving 1 g each of AG, CMS, and Qch in 76.66 mL of DDW. These sponges also need to be cross-linked and lyophilized to obtain the final sponges.

### 2.3 Physicochemical characterizations

#### 2.3.1 FTIR spectroscopy

The FTIR spectrometer (Thermo Fisher Scientific, Waltham, MA, United States) was used to measure the infrared spectra of sponges in the wavelength range of 500–3,500 cm^−1^.

#### 2.3.2 SEM

By using SEM (VEGA3, TESAGN, Czech Republic) with an acceleration voltage of 10 kV, the porous morphology of the sponges was examined. To calculate the pore size, Image J software was used to analyze the SEM images. The pore size was calculated from measurements of more than 30 pores taken from different SEM images.

#### 2.3.3 Porosity test

The volume (V) was obtained by measuring the diameter and height of the sponge, and then the pre-weighed sponge (m_0_) was entirely submerged in anhydrous ethanol and the weight was recorded as m_1_ after removing the excess ethanol on the surface. The porosity was calculated by the following formula:

$$Porsity=\frac{m_{1}-m_{0}}{\rho V}$$

where ρ is the density of anhydrous ethanol.

#### 2.3.4 Fluid absorption rate measurement

The pre-weighed sponge (m_0_) was placed on the slides and phosphate buffer solution (PBS) was added drop by drop to the sponge until the sponge reached equilibrium. Excess liquid was removed from the sponge surface with filter paper and the weight of the wet sponge was recorded as m_1_. The fluid absorption ratio was calculated as below:

$$Fluid\;absorption\;rate=\frac{m_{1}-m_{0}}{m_{0}}\times 100\%$$

#### 2.3.5 WVTR

Dried sponges of equal size were placed on the mouth of a cylindrical bottle containing 10 mL of DDW and tightly covered with plastic wrap to prevent additional evaporation of water. The weight of water absorbed by the sponge after 24 h was measured and the WVTR was calculated as follows:

$$WVTR=\frac{W_{i}-W_{t}}{S}g/m^{2}/day$$

where W_i_, W_t,_ and S are the weight of the bottle containing, the weight of the bottle containing water after 24 h, and the area of the mouth of cylindrical bottles, respectively.

### 2.4 *In vitro* cytocompatibility assay

The Cell Culture Centre of Shanghai Institutes for Biological Science Chinese Academy of Sciences (Shanghai, China) contributed the mouse fibroblast cells (L929). L929 cells were incubated at 37°C in 5% CO_2_ and the medium was changed every 2 days.

The cell live/dead assay kit (Meilunbio, China) and CCK-8 assay kit (MedChem Express Co., Ltd., China) were used to assess the sponge’s cytotoxicity. For live/dead staining, L929 cells were grown in 24-well plates at a density of 1 × 10^4^/well. 12 h later, equal amounts of sterile sponges were added to each well separately. After co-culture with L929 cells overnight, the sponges were removed. Cells were then washed with PBS and incubated for 30 min at 37°C with the addition of 2.5 µm calcein-AM and 2.5 µm ethidium homodimer protected from light. After rinsing again with PBS, images of the cells were acquired by fluorescence microscopy (Nikon A1 MP, Japan), and the proportion of living cells was calculated. As for the CCK-8 assay, L929 cells were planted on 24-well plates at a density of 2 × 10^3^/well. 12 h later, the cells were treated with different sponges. The sponges were co-cultured with the cells for 1–5 days, respectively. The sponges were removed, and the cells were rinsed with PBS. Cells were then cultured in a medium supplemented with 10% CCK-8 solution and the optical density (OD) value was measured at 450 nm by a microplate reader (Bio-Tek, United States).

### 2.5 *In vitro* antibacterial activity test

*Escherichia coli* (*E. coli*, Gram-negative), *Staphylococcus aureus* (*S. aureus*, Gram-positive), and *Candida albicans* (*C. albicans*) were cultured with LB Broth (Solarbio, China), Tryptic Soy Broth (Solarbio, China), and Brian Heart Infusion (Solarbio, China), respectively. Equal masses of sponges were co-incubated with 4 mL 10^6^ CFU/mL bacterial suspension for 12 h. The control group was the normal bacterial solution without the addition of samples. The co-cultured bacterial solution was diluted and 20 μL suspension was evenly spread on agar plates. After incubation at 37°C for 18 h, the microbial colonies formed on the agar plates were counted.

### 2.6 Blood coagulation test

Different composite sponges and AGS (Xiangen Medicine, China) were prepared to the same size and placed on Petri dishes. The AGS was used as a positive control (+) and fresh blood was used as a negative control (−). Next, the sponge was then loaded with 200 μl of whole blood and incubated at 37°C for 5 min. And 5 mL DDW was added carefully to the Petri dish to remove the unclotted blood. Coagulation of the blood on the sponge was observed. Subsequently, the OD value of the solution containing hemoglobin was measured at 540 nm with a microplate reader to assess the coagulation capacity of the sponge.

### 2.7 Hemolysis assay

The whole blood was donated by healthy volunteers according to the relevant guidelines and used after dilution five times. 10 mg of the sponge was added to 1 mL of PBS, followed by 50 μl of diluted blood. DDW and PBS solution was used as the positive control (+) and negative control (−), respectively. After thorough mixing, the samples were incubated in a 5% CO_2_ incubator at 37°C for 1 h and then centrifuged at 1,500 rpm for 5 min. The absorbance of the supernatant at 540 nm was recorded using a microplate reader. The hemolysis ratio was calculated with the formula below:

$$Hemolysis\;\%=\frac{{OD}_{sponge}-{OD}_{\left(-\right)}}{{OD}_{\left(+\right)}-{OD}_{\left(-\right)}}\times 100\%$$

### 2.8 Red blood cell (RBC) adhesion

Petri dishes were used to place AGS and composite sponges. 200 μl of whole blood containing 10% sodium citrate was added dropwise to the sponges and incubated at 37°C for 30 min. All samples were carefully rinsed with PBS solution to remove the unadhered RBC. Sponges with agglutinated RBC on the surface were fixed by 2.5% glutaraldehyde for 2 h. The samples were then dehydrated with different concentrations of alcohol solutions and further observed by SEM.

### 2.9 *In vivo* hemostatic assessment

#### 2.9.1 Hepatic trauma model of mouse

A mouse model of liver injury was used to perform *in vivo* hemostasis studies. 8-week male mice were selected for the surgery. The mouse was anesthetized with sodium pentobarbital and fixed on a wooden board. A linear wound with a depth of 1 mm and length of 5 mm was created in the liver and the lost blood was collected with a pre-weighed filter paper located below the liver. The composite sponges and AGS (14 mm × 14 mm × 5 mm) were quickly placed on the wound site. The untreated liver wound was used as a negative control. The hemostasis of the liver was photographed and recorded at specific time points, and the filter paper was weighed to calculate the amount of blood loss.

#### 2.9.2 Tooth extraction model of pig

The tooth extraction model was used to evaluate the hemostasis effect of the composite sponges on post-extraction bleeding. 6-week male mini pigs were selected for the surgery. After anesthesia, the mini pig was secured to the operating table and the mouth was pulled apart to expose the maxillary second primary molar. The gingiva was carefully separated with a dental probe. After extraction, the socket was filled with the ACQ-2 sponge or AGS to stop the bleeding. Sterile gauze was used to absorb the spilled blood. The time of hemostasis and the amount of blood loss were recorded as described in 2.9.1. All the experiments were approved by the Ethics Committee of the Affiliated Hospital of Qingdao University (approval number: QYFY WZLL 27368).

### 2.10 Statistical analysis

All data were presented as mean ± standard deviation. GraphPad Prism eight software was used for statistical evaluation. To determine differences between groups, one-way analysis of variance (ANOVA) was used to determine statistical significance, and a value of *p* < .05 was considered to be statistically significant.

## 3 Results and discussion

### 3.1 Preparation and characterization of composite sponges

**FIGURE 1:**
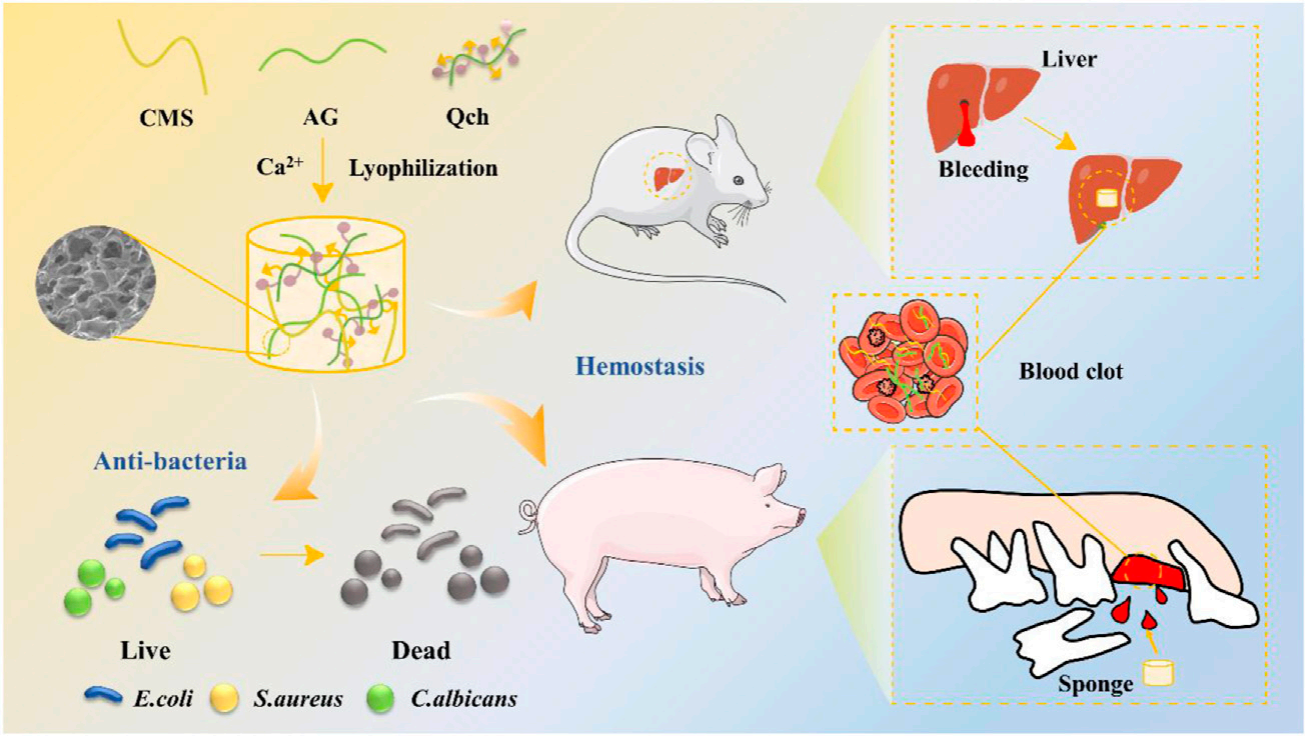
Schematic diagram of the synthesis of ACQ sponges and their application in antibacteria and hemostasis.

The strategy to develop ACQ composite sponges with enhanced hemostatic and antibacterial performances is outlined in Figure 1. The porous ACQ sponges were fabricated *via* Ca^2+^ crosslinking, electrostatic interaction, and lyophilization, which possess great promise to be a hemostatic material for avoiding dry socket following tooth extraction.

**FIGURE 2:**
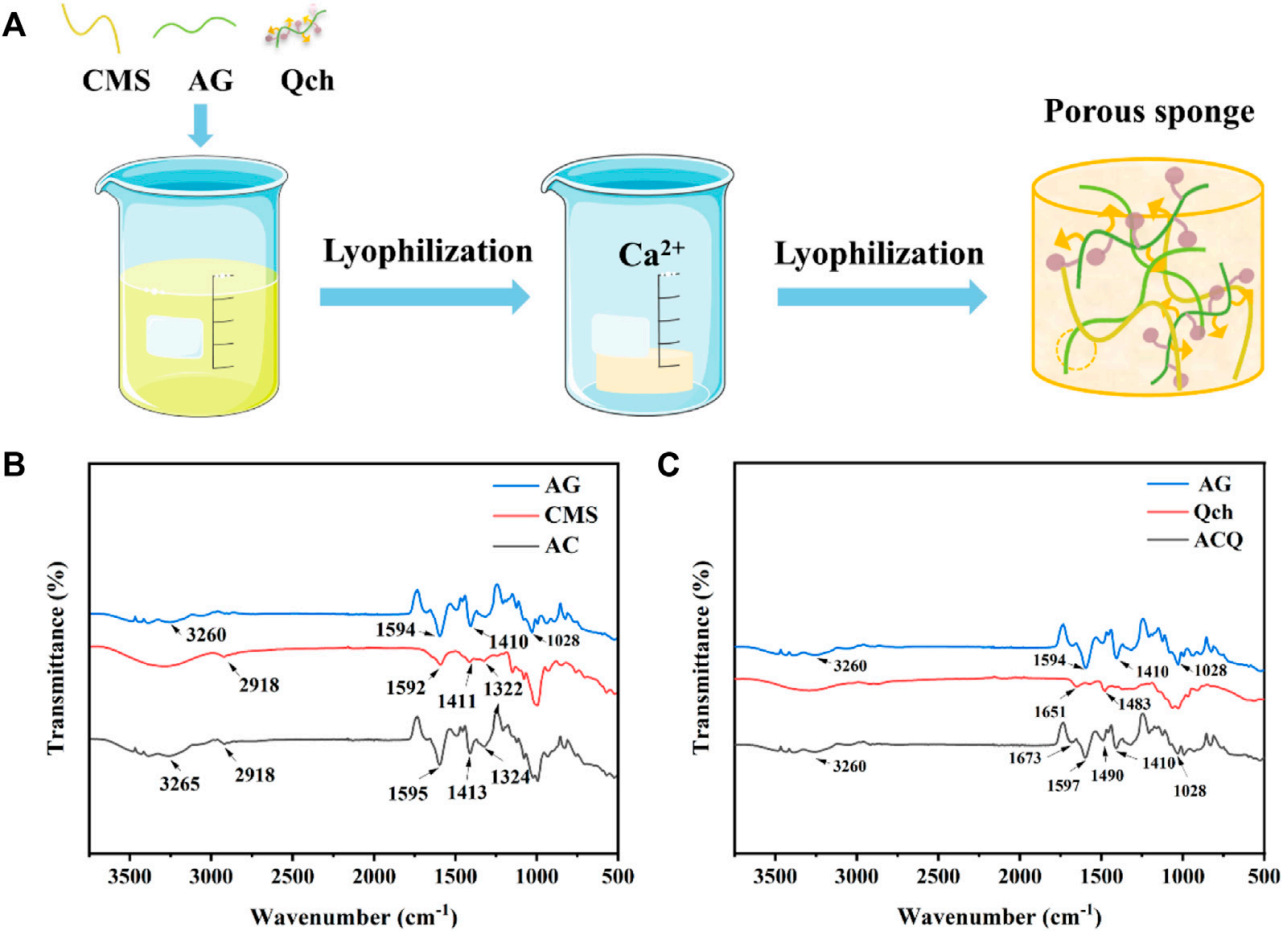
**(A)** Schematic diagram of the synthesis of ACQ sponges. **(B)** FTIR spectra of AG, CMS, and AC. **(C)** FTIR spectra of AG, Qch, and ACQ.

ACQ composite sponges were developed as a hemostatic material *via* Ca^2+^ crosslinking, electrostatic interaction, and lyophilization procedures for wound management after tooth extraction as shown in Figure 2A–C show the FTIR spectra of AG, CMS, Qch, and various sponges. The prominent peaks at 3,260, 1,594, 1,410, and 1,028 cm^−1^ in the AG were generated by the stretching vibration of -OH, symmetric and asymmetric stretching vibrations of the carboxylate groups, and the vibration of the COC group, respectively (Zhao et al., 2016). For the CMS, there were obvious absorption peaks at 2,918 and 1,592 cm^−1^, which were assigned to the tensile vibration of C-H bonds in methylene and modification of starch by carbonyl functional groups, respectively. The transmittance bands at 1,411 and 1,322 cm^−1^ belong to -CH_2_ stretching vibration and -OH bending vibration, respectively (You et al., 2016). The spectra of AC revealed the characteristic bands of both AG and CMS. Qch showed two characteristic absorption peaks of 1,651 and 1,483 cm^−1^, which correspond to the vibration of amide I and the introduction of quaternary ammonium groups on the chitosan, respectively (Li et al., 2010). ACQ preserved these feature peaks, illustrating the successful preparation of the composite sponge.

**FIGURE 3:**
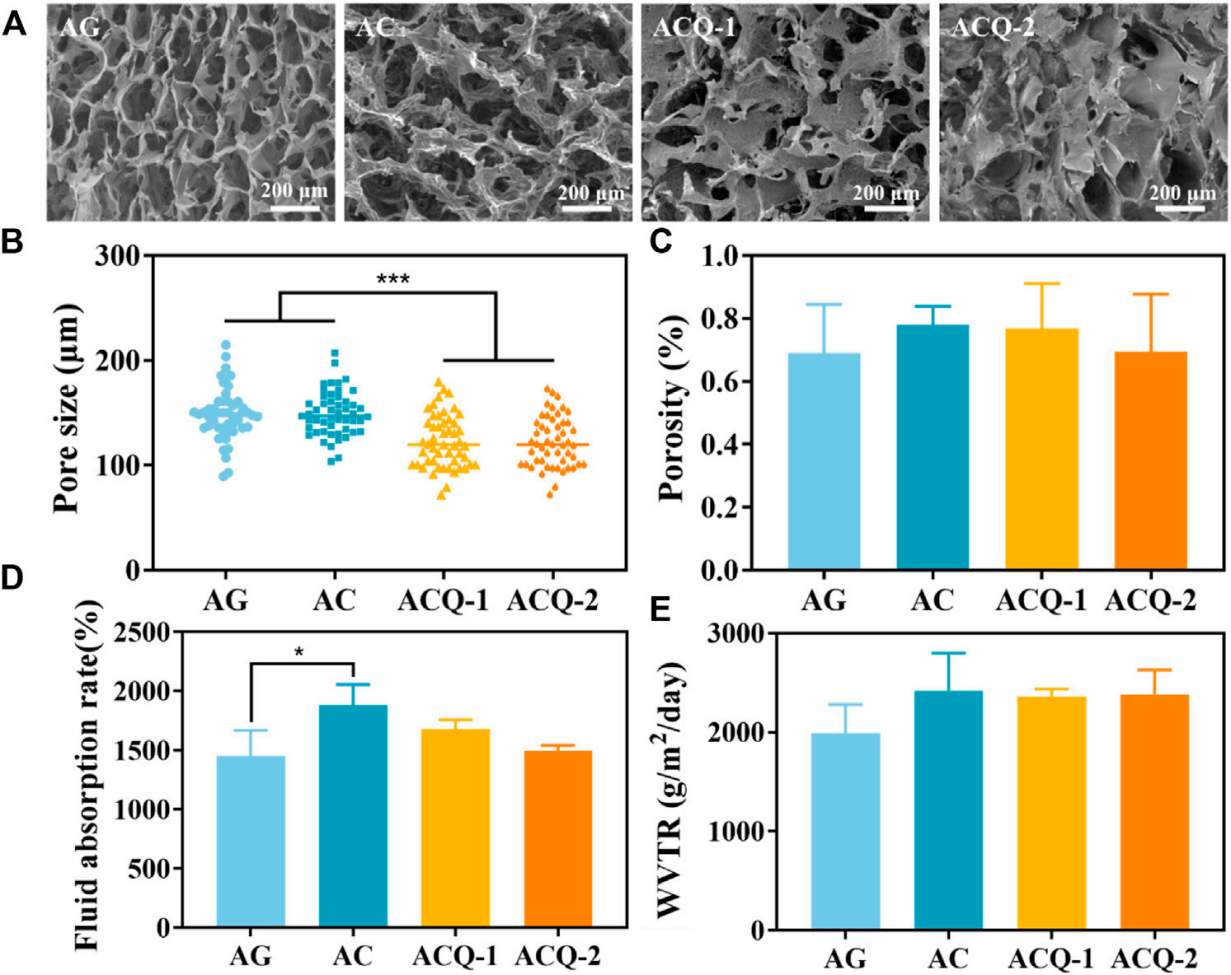
**(A)** SEM images of AG, AC, ACQ-1, and ACQ-2 sponges at ×100 magnifications, respectively. **(B)** Pore size, **(C)** porosity, **(D)** fluid absorption rate, and **(E)** WVTR of various sponges. Data are mean ± SD (n = 3) (*p < 0.05, ***p < 0.001).

The internal microstructure of the sponges was observed by SEM (Figure 3A). Each group of sponges exhibited an interconnected porous structure with an irregular arrangement. As shown in Figure 3B, the pore size of ACQ-1 and ACQ-2 sponges was significantly smaller than that of AG and AC sponges due to the addition of Qch. However, there was no significant difference in porosity among the various sponges (Figure 3C). The high specific surface area and hierarchically porous structure of composite sponges could vastly improve the contact area with blood, accelerate hemostasis and absorb wound exudate, as well as promote trauma healing (Yan et al., 2022). High liquid absorption capacity can effectively absorb tissue exudate and achieve rapid hemostasis (Shen et al., 2022). As depicted in Figure 3D, the fluid absorption rate of the AC sponge was higher than that of the AG sponge, but not significantly different from that of the ACQ-1 and ACQ-2 sponges. The high fluid absorption rate could enhance the sponge’s ability to absorb blood, which is closely related to the pore size and specific surface area (Bian et al., 2022). Meanwhile, the polysaccharide chain of CMS contains a large number of hydrophilic groups, and the O-H and -COOH groups could enhance the attraction to water molecules (Yusof et al., 2018). Proper infiltration of water and gas could accelerate wound healing while promoting blood absorption in the wound dressing (Yang Y. et al., 2022). There was no significant difference in WVTR among various sponges as WVTR is mainly related to porosity (Figure 3E).

### 3.2 Cytocompatibility evaluation of composite sponges

**FIGURE 4:**
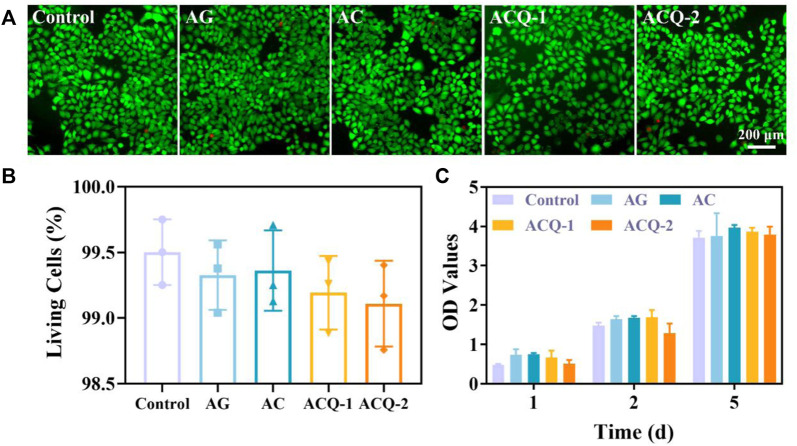
**(A)** Fluorescent images of cell live/dead staining of fibroblasts, and **(B)** the rate of living cells after co-cultivation with various sponges for 24 h, respectively. **(C)** The cytotoxicity of sponges after incubation with L929 cells for 1–5 days. Data are mean ± SD (n = 3).

The hemostatic material should possess good biocompatibility to avoid delaying wound healing. Various sponges were co-cultured with L929 cells to assess their cytocompatibility using cell live/dead staining and CCK-8 assay, as fibroblasts are vital for wound healing (He et al., 2021; Mei et al., 2021; Hao et al., 2022a). The live/dead staining displayed in Figure 4A demonstrated that there were no significant differences in cell number and cell viability among sponges, and the cell viability was all higher than 98.5% (Figure 4B). As detailed in Figure 4C, the number of cells increased steadily from 1–5 days, indicating that the composite sponge promoted the cell proliferation which could contribute to the wound healing. This may be due to the fact that AG, CMS, and Qch have been proven to possess desirable biocompatibility and are widely used in biomaterials and tissue engineering (Jin et al., 2018; Yusof et al., 2018).

### 3.3 Antibacterial ability of the composite sponges

**FIGURE 5:**
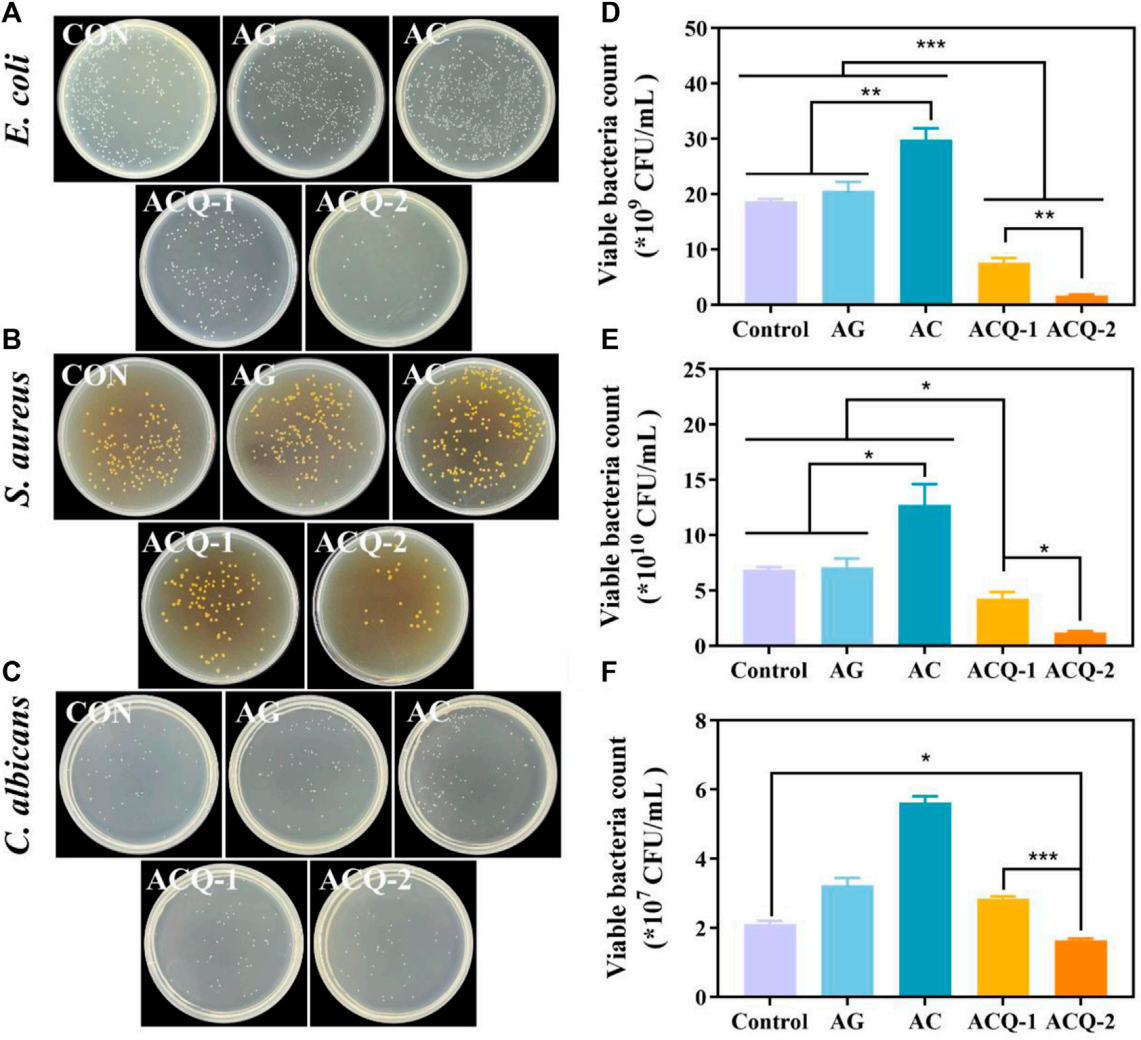
**(A–C)** Bacterial and fungus colonies, and **(D–F)** quantitative analysis of viable bacteria of *E. coli*, *S. aureus*, and *C. albicans* on agar plates after being co-cultured with the sponges, respectively. Data are mean ± SD (n = 3) (*p < 0.05, **p < 0.01, ***p < 0.001).

The effect of composite sponges against *E. coli*, *S. aureus*, and *C. albicans* was tested by CFU count test. As illustrated by Figure 5A,B, the colonies in ACQ-1 and ACQ-2 sponge groups were significantly reduced due to the incorporation of Qch. Moreover, the effect of inhibiting bacteria was significantly enhanced with the increase of Qch content (Figure 5D,E). This may be due to the fact that quaternary ammonium salts could alter the permeability of the bacterial cell wall and reduce the exchange of bacterial nutrients, leading to protein denaturation and ultimately cell death (Cheah et al., 2019). As for *C. albicans*, only the ACQ-2 sponge effectively inhibited the growth of the fungus (Figure 5C,F). The interaction of CS cationic chains with negatively charged macromolecular residues on the fungal cell membrane, which results in death by allowing internal electrolytes and other components to leak out, may be the cause of the antifungal effects of Qch (Lee et al., 2018). Quantitative results showed that the AG, AC, and ACQ-2 groups could not inhibit the growth of bacteria and fungi. This may be because fungi are eukaryotes and are more difficult to eliminate than bacteria (Campoy and Adrio, 2017). Thus, only ACQ-2 with the highest Qch content had a slight antifungal effect.

### 3.4 Blood coagulation and hemocompatibility tests of composite sponges

**FIGURE 6:**
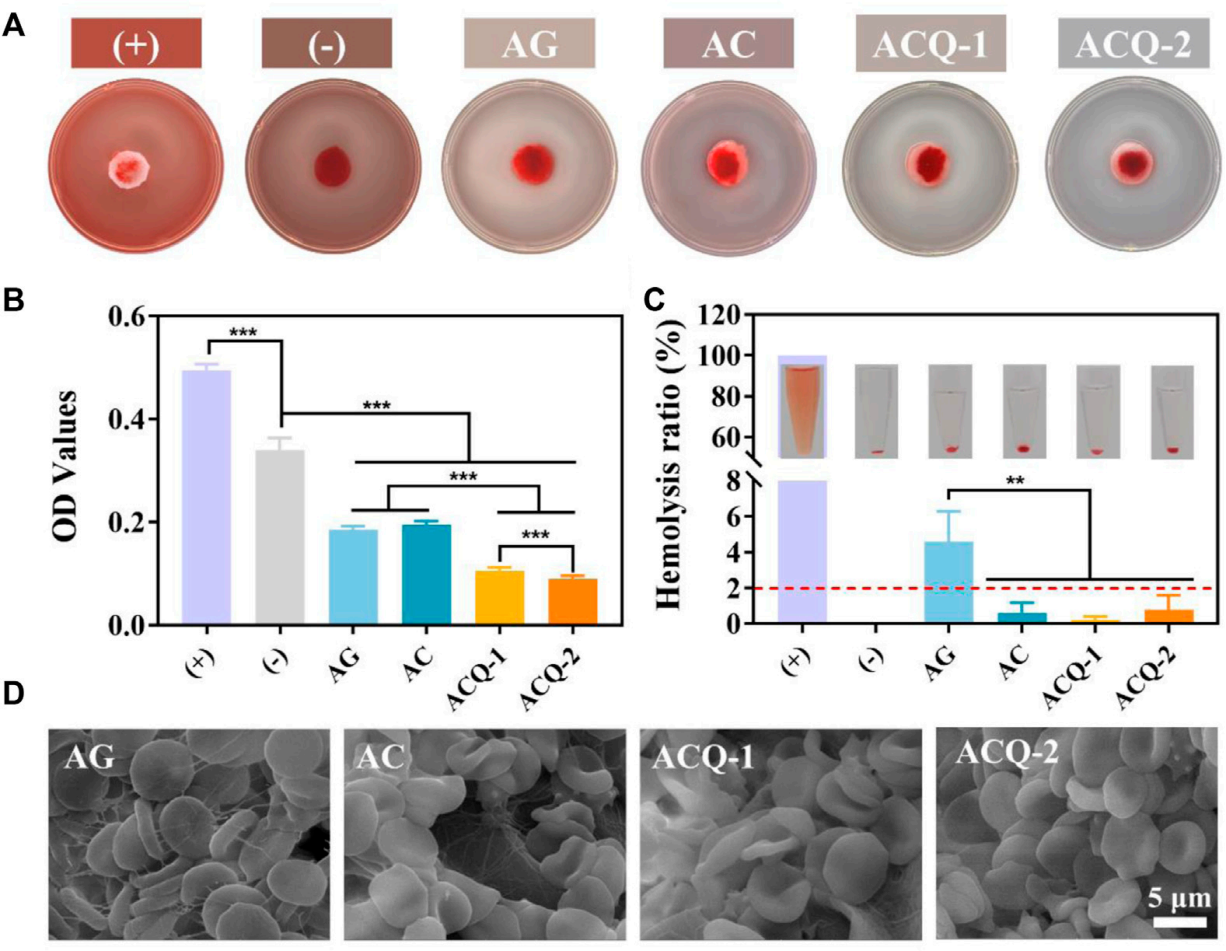
**(A)** Pictures and **(B)** quantitative results of blood coagulation experiments. **(C)** Photographs and quantification of hemolysis test. **(D)** SEM images of RBC adhering to sponges. Data are mean ± SD (n = 3) (**p < 0.01, ***p < 0.001).

An excellent hemostatic material should be able to promote blood clotting. The hemostatic effect of sponges was verified by detecting the formation of blood clots. As displayed in Figure 6A, after rinsing with DDW, the AGS and (-) control groups showed a red color due to the uncoagulated blood, while the developed sponge groups were significantly lighter in color. Meanwhile, the area of blood stains on the sponges of the AC, ACQ-1, and ACQ-2 groups was smaller than that of the AG group, which also indicates that the designed sponges possessed the ability of fast hemostasis. Figure 6B exhibits the quantitative results of blood coagulation experiments. Interestingly, the coagulation-promoting effect of AGS appeared to be very limited, and its OD value was even higher than that of the (-) control group. It can be observed that both AG and AC sponges effectively promoted the formation of blood clots, which is related to the fact that AG and CMS could promote fibrinogen adsorption and platelet aggregation (Chen et al., 2018; Shen et al., 2022). The ability of the sponge loaded with Qch to promote clotting was further enhanced. The positive charge on the sponge surface could adhere to more RBCs due to electrostatic interactions as shown in Figure 6D (Xu G. et al., 2022; Wei et al., 2022). Moreover, an excellent hemostatic material should be hemocompatible while promoting clotting. The hemolysis experiments revealed that the hemolysis rate of all groups of sponges was less than 2% (Figure 6C), and the RBC adhering to the sponges all showed a typical biconcave disc shape (Figure 6D). These results indicate that the synthetic composite sponges are highly desirable materials for trauma treatment after tooth extraction and the prevention of dry socket.

### 3.5 *In vivo* hemostatic activity of sponges

**FIGURE 7:**
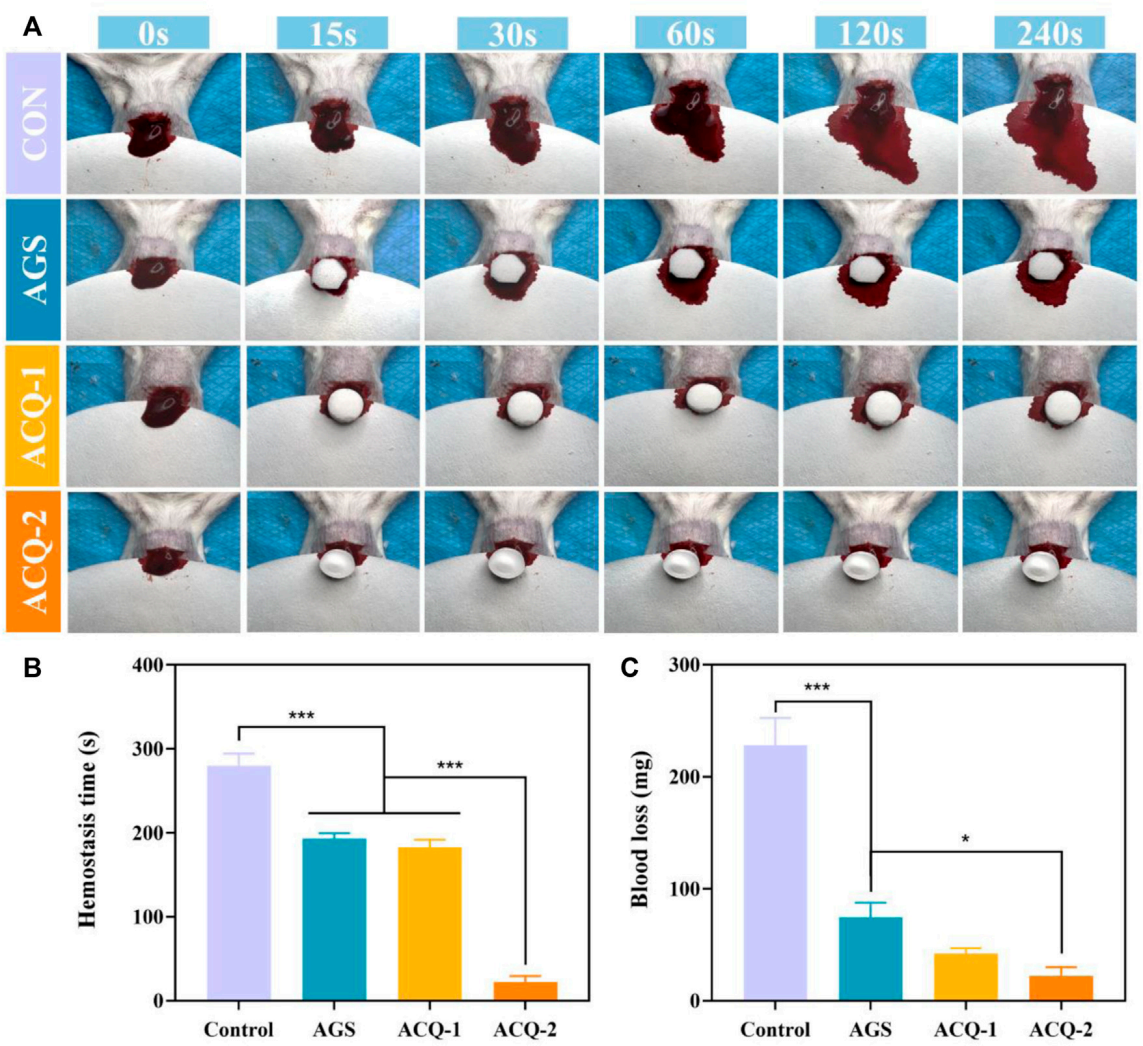
**(A)** Photographs of blood flow taken at specific times during liver hemostasis experiments. **(B)** Blood loss and **(C)** hemostasis time in liver hemostasis experiments. Data are mean ± SD (n = 3) (*p < 0.05, ***p < 0.001).

The formation of blood clots rich in growth factors can protect the tooth extraction wound to prevent the occurrence of dry socket, and also plays an important role in mediating the early inflammatory response, leading to the acceleration of tissue repair and regeneration (Wu et al., 2022). The hemostatic effect of composite sponges *in vivo* was further evaluated by a mouse liver injury model. Figure 7A depicts the coagulation process in the injured liver. It was found that blood loss has been increasing rapidly in the untreated control group. As shown in Figure 7B, blood loss was significantly reduced with AGS and composite sponges, and the hemostatic effect of composite sponges was more pronounced. In terms of hemostasis time, ACQ-2 achieves effective hemostasis in a very short period (Figure 7C).

**FIGURE 8:**
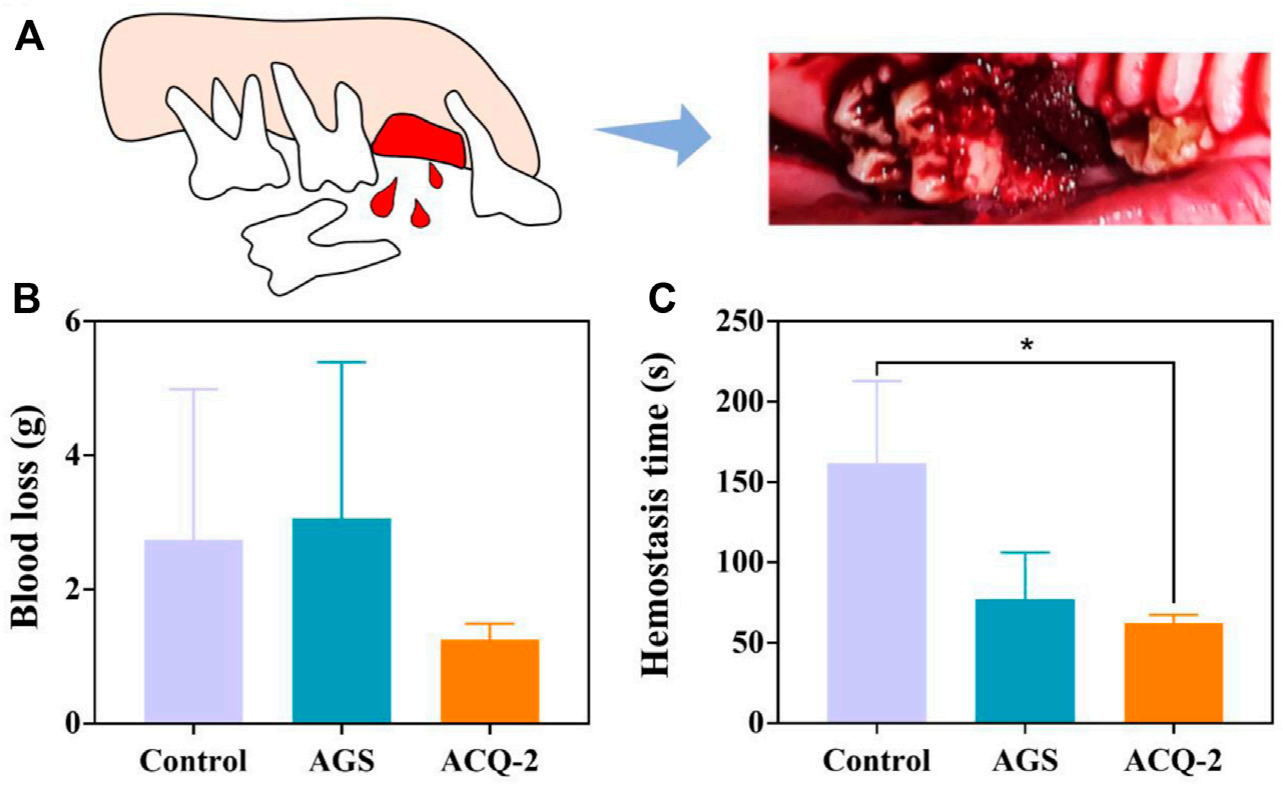
**(A)** Schematic diagram of pig tooth extraction. **(B)** Blood loss and **(C)** hemostasis time in tooth extraction hemostasis experiments. Data are mean ± SD (n = 3) (*p < .05).

Based on the above experimental results, the ACQ-2 sponge was used to perform hemostasis experiments for tooth extraction in mini pigs. Due to individual differences in the pigs and the effect of the extraction operation on blood loss, there was no statistical difference in blood loss among the control, AGS, and ACQ-2 groups. However, the ACQ-2 sponge group had the least mean blood loss. Moreover, the hemostasis time of ACQ-2 was almost halved relative to the control group, indicating that it accelerated the formation of blood clots in the extraction sockets. As mentioned above, this may be due to the fact that the positive charge on Qch could attract the negative charge on the erythrocyte surface through electrostatic interaction, thus promoting the aggregation of RBC. Meanwhile, the Ca^2+^ contained in the cross-linker can activate endogenous and exogenous coagulation pathways, enriching more RBC and promoting blood coagulation Figure 8.

## 4 Conclusion

In summary, bio-multifunctional AG/CMS/Qch sponges with excellent antibacterial and rapid hemostatic properties were successfully fabricated by Ca^2+^ cross-linking, electrostatic interaction, and lyophilization methods for hemostasis of tooth extraction wound and prevention of postoperative dry socket. It was found that the developed sponges with porous structures possess a high fluid absorption rate and suitable WVTR to speed up the hemostasis process. Furthermore, biological experiments suggest that the fabricated sponges exhibited satisfactory biocompatibility and a distinctly antibacterial effect. Additionally, sponges could absorb blood and promote the aggregation of RBC to achieve rapid hemostasis. Therefore, the bio-multifunctional sponges with satisfactory antibacterial properties and significant rapid hemostatic effect can be performed as a potential novel type of clinical hemostatic products for trauma treatment after tooth extraction and prevention of dry socket.

## Data Availability

The original contributions presented in the study are included in the article/supplementary material, further inquiries can be directed to the corresponding authors.
